# Disseminated cryptococcosis initially presenting as cellulitis in a patient suffering from nephrotic syndrome

**DOI:** 10.1186/1471-2369-14-20

**Published:** 2013-01-22

**Authors:** Wentao Ni, Qi Huang, Junchang Cui

**Affiliations:** 1 Department of Respirology, Chinese PLA General Hospital, No.28, FuXing Road, Beijing, China; 2 Department of Nephrology, Chinese PLA General Hospital, No.28, FuXing Road, Beijing, China

**Keywords:** Cutaneous cryptococcosis, Cellulitis, Nephrotic syndrome, Immunosuppression

## Abstract

**Background:**

Cryptococcosis is considered as an opportunistic infection because it mainly occurs in immunosuppressed hosts. C. neoformans is usually acquired by the respiratory route and then may disseminate hematogenously to other places, such as meninges, bone and skin. Cutaneous involvement is a rare but important feature of disseminated cryptococcosis with a poor outcome if misdiagnosis. We reported the first case of patients with nephrotic syndrome suffering from disseminated cryptococcosis initially presented as cellulitis.

**Case presentation:**

A 34-year-old man developed severe cellulitis on his both lower extremities without any preceding injury and allergies. The patient had been treated with systemic corticosteroids nearly one year for nephrotic syndrome. According to the outcome of blood culture, the wound area was interpreted as bacterial cellulitis at first. However, the antimicrobial treatment made no response and the skin biopsy revealed the presence of Cryptococcus neoformans, which was subsequently confirmed by microbiological culture. Though the initiation of therapy with fluconazole 400 mg per day was immediately adopted, the patient’s conditions suddenly plummeted and he died in the end.

**Conclusion:**

Since the poor outcome of disseminated cryptococcosis if unrecognized and untreated in time, it should be investigated rigorously as a differential diagnosis in patients with nephrotic syndrome suffering from cutaneous diseases.

## Background

Cryptococcus neoformans, a non-mycelial encapsulated budding yeast characterized by producing a teleomorph state (the sexual stage) and an anamorph state (asexual reproductive stage), are important opportunistic pathogens that usually infect immunosuppressed individuals [1]. The most common exposure sources are inhalation of aerosolized infectious yeast from pigeon, avian excreta, contaminated soil, milk, fruits and wood products [2,3]. These organisms then may hematogenously disseminate to other places, mainly the central nervous system [4]. cryptococcal skin disease is a rare feature of disseminated cryptococcosis, and has a poor outcome if unrecognized and untreated [5]. Here, we present a case of cryptococcal cellulitis in a patient with nephrotic syndrome who was receiving long-term steroid treatment. Reviewing the literature, this is the first report of nephrotic syndrome with disseminated cryptococcosis initially presented as cellulitis.

## Case presentation

**Figure 1:**
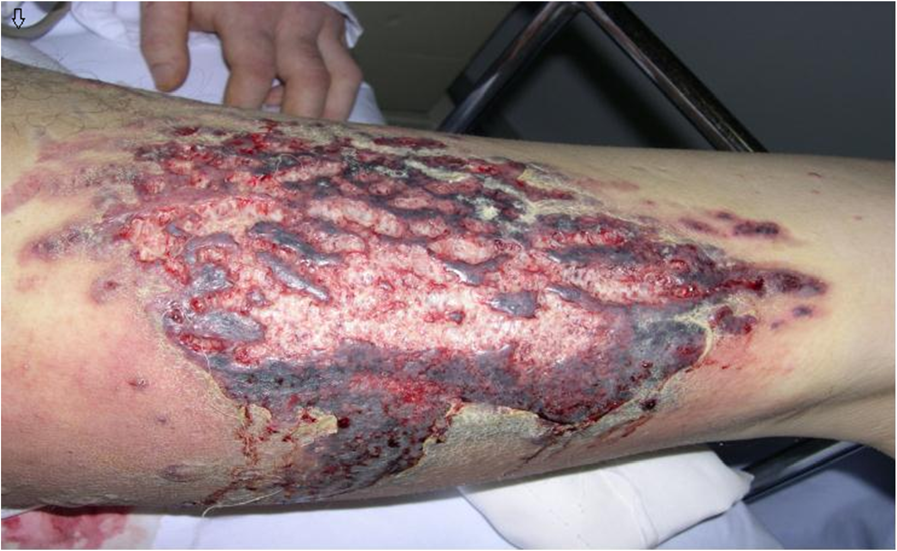
Massive shallow ulcers on the left inner thigh surrounded by reddish, infiltrated steak.

A 34-year-old man was admitted to our hospital presented with massive shallow ulcers of both lower extremities (Figure 1), fever of 39°C and severe pain. One year ago, he was diagnosed with nephrotic syndrome and the renal biopsy which showed the pathological type was minimal change glomerulopathy. After a treatment with 60 mg of prednisolone and 20 mg of furosemide daily, his Lower extremity edema gradually disappeared and the proteinuria became negative. However, 3 months later, the edema occurred again due to the reduction of dose step by step. In order to restrain the development of the disease, he was treated with 48 mg of Triamcinolone daily at local hospital. Before long, he presented with high fever, skin ulceration and severe pain of his Lower extremities without history of allergies or close contact to any animals. Although he had received antibiotics in another hospital, the cutaneous lesions gradually enlarged and sustained high fever continuously existed.

On examination, the both inner thigh exhibited massive necrotic superficial ulceration overlying erythema, each approximately 20 cm in diameter; the forearms exhibited several reddish indurations. No regional lymphnodes were involved. Urinalysis revealed proteinuria (5 g/L) and haematuria (15/HPF). The 24 h urine protein was 4.15 g and the plasma albumin was 24.7 g/L. The total serum cholesterol and serum triglycerides were 7.56 mmol/L and 3.02 mmol/L, respectively. Blood urea nitrogen and creatinine were 100 mg/dl and 12.5 mg/dl, respectively. In addition, the anti-HIV antibodies, anti-citrullinated peptide antibodies (ACPA), anti-nuclear antibodies (ANA), and anti-neutrophil cytoplasmic antibodies (ANCA) were all tested negative. The initial chest X-ray revealed a normal finding.

On the day of admission, we initiated an empirical treatment with moxifloxacin and cefmenoxime intravenously due to the assumed bacterial cellulitis. In addition, Gamma globulin was added for enhancing his immunity and 60 mg of prednisolone daily was still administrated for the nephrotic syndrome. Blood cultures were obtained in order to clarify the pathogen. Considering the potential inflammation dissemination caused by skin biopsy, we did not do it initially.

Three days later, the cutaneous lesions had slightly reduced with the high fever continuous existence. The outcome of blood culture was coagulase negative staphylococci and the X-ray of the lung revealed a change of ground-glass opacity in both perihilar regions. Treatment regimen changed to Linezolid plus Caspofungin through the outcome of blood culture. However, the patient has little response to the treatment with the appearance of gradually blurred consciousness, delirium, and hypoxemia. Then he was transferred to respiratory intensive care unit for further therapy.

**Figure 2:**
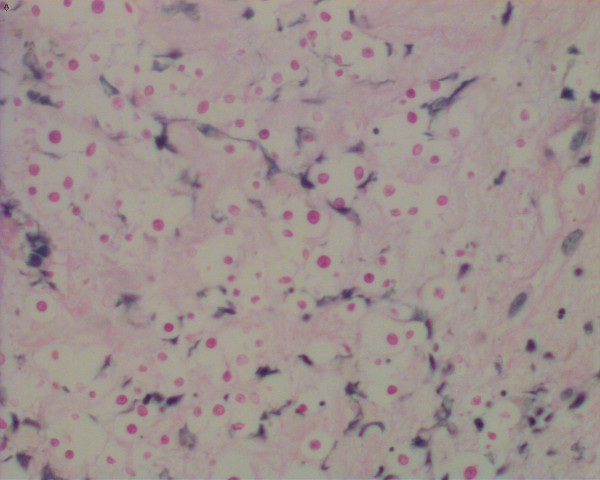
**Light microscopic histological findings of the skin biopsy specimen from the cutaneous lesion showed an inflammatory infiltration containing PAS positive micro-organisms.** (Periodic acid-Schiff stain; original magnification: ×400).

In the end, investigations on the biopsy revealed the existence of C. neoformans after four days of admission (Figure 2) and the capsular polysaccharide antigen titer of C. neoformans in blood determined by latex particle agglutination method was 1:1024. Microbiological cultures of the biopsy specimen, blood and sputum of the patient subsequently verified the infection of C. neoformans. Although the patient became unconscious indicating a probability of central nervous system involvement, his family refused lumber puncture. Antimycotic therapy with intravenous fluconazole (400 mg daily) was immediately adopted and the empirical intravenous antibiotic therapy continued.

Unfortunately, on the 6th day, the patient suddenly appeared the signs of acute respiratory failure with intractable hyoxemia and the mechanical ventilation was initiated. But then acute heart failure occurred. Despite timely rescue, his blood pressure was still at a quite low level maintained by dopamine accompanied with oliguria. The patient died on the eight day of admission.

## Conclusions

Immunologic impairment can frequently occur in patients with nephrotic syndrome because of low serum immunoglobulin G concentrations, reduced complement activity, depressed T-cell function and the use of corticosteroids or cytotoxic agents [6]. The immunologic impairment, adding the protein-rich edematous skin serving as a good media, increases the vulnerability to various cutaneous infections [7] (be reported in up to 27.02% [8]), particularly the cellulitis. The most common pathogens that cause cellulitis are β-hemolytic Streptococci and some gram-negative bacteria such as E.coli [9]. But other atypical organisms shouldn’t be neglected considering the immunocompromised condition in nephrotic syndrome patients just as our case.

As important opportunistic pathogens, C. neoformans usually infect individuals with AIDS. Nevertheless, with an increasing number of steroid-dependent individuals as our patient in the last decade, the prevalence rate of cryptococcosis is continuously rising [10]. In humans, C. neoformans usually cause three types of infections: pulmonary cryptococcosis, cryptococcal meningitis and wound or cutaneous cryptococcosis [11]. Cutaneous involvement, a rare but important feature of disseminated cryptococcosis, may precede systemic symptoms by as long as 8 months [12]. Naoko Ogami has report a patient with nephrotic syndrome suffering from primary cutaneous cryptococcosis without other organ involvement [13]. However, cutaneous cryptococcal disease usually represents the hematogenous dissemination of cryptococcosis. Its presentation has various clinical morphologies: a draining sinus tract, verrucous nodules, molluscum-like lesions and erythematous, indurated plaques, and so on [14]. Yet, none of these manifestations are pathognomonic for cryptococcal infection. Confusion with other resemble diseases is not uncommon and a diagnostic delay might be a major factor contributing to high associated mortality [5]. A timely histological analysis of skin biopsies can assist to confirm the pathogens. In addition, the importance of CSF cultures and serological studies should not be over emphasized [15].

According to the current guidelines of the Infectious Diseases Society of America (IDSA), amphotericin B combined with flucytocine is recommended as primary therapy regimen for disseminated cryptococcosis followed by fluconazole as consolidation therapy [16]. To those with renal dysfunction, lipid formulations of AmB or AmB lipid complex could replace Am B to avoid further deterioration of the renal function. With appropriate systemic antifungal therapy, 74% of patients with cryptococcal cellulitis can be expected to survive in immunocompetent status [17]. However, to those having developed into organ failures, as seen in our patients, a review of the literature suggests a much graver prognosis. The mortality rate of ARF result from disseminated cryptococcosis was 100% and 55% respectively in immunocompromised patients with or without AIDS [5,18], and nearly all deaths occurred within 2 weeks of diagnosis with or without treatment indicating an aggressive and fulminant progress of the disease.

There are several potential explanations for the poor prognosis of our patients. First, a severe disseminated cryptococcal infection that caused multiple organ failure lead to the poor outcome. Second, the delayed diagnosis and antifungal therapy have also been a contributing factor. Finally, the patients’ suddenly deteriorated condition after two day’s antifungal therapy reminded that immune reconstitution inflammatory syndrome (IRIS) might also have contributed to his death. The IRIS which manifests with inflammatory reactions, targeted at the antigens of opportunistic infections can occur in immunocompromised patients after some degree of immune restoration [19]. If the IRIS occurs in patients already diagnosed with opportunistic infections, it may result in recurrence or worsening of clinical features despite effective treatment [20]. It has been reported that in patients with cryptococcal meningitis, antifungal therapy may prompt the reversion of an immunosuppressive Th2 response to the normally protective Th1 cytokine response, leading to an exuberant host response against residual sites of disease [21]. Because we didn’t monitor the changes of T cells in our patient, the IRIS as a risk factor can’t be excluded completely.

In conclusion, this case demonstrated that C. neoformans should be considered in the differential diagnosis of cutaneous infection in nephrotic patients or other immunosuppressive patients. Even when the organism is clear, one can't be too cautious to reconsider the potential existence of other atypical pathogens if the initial treatment makes little effects.

## Competing interests

The authors declare that they have no competing interests.

## Authors’ contributions

All the authors were involved in the clinical management of the case and the writing of the report. All authors read and approved the final manuscript.

## Pre-publication history

The pre-publication history for this paper can be accessed here:

http://www.biomedcentral.com/1471-2369/14/20/prepub
